# Hashimoto’s Encephalopathy Mimicking Viral Encephalitis: A Case Report

**DOI:** 10.3389/fnins.2020.00331

**Published:** 2020-04-15

**Authors:** Miaomiao Yu, Yu Yang, Xianyi Ma, Yinyin Xie, Ningning Sun, Hongmei Meng

**Affiliations:** Department of Neurology and Neuroscience Center, The First Hospital of Jilin University, Changchun, China

**Keywords:** Hashimoto’s encephalopathy, viral encephalitis, differential diagnosis, anti-thyroid antibodies, therapy

## Abstract

Hashimoto’s encephalopathy (HE) is a rare neuropsychiatric syndrome characterized by elevated levels of anti-thyroid antibodies. Diverse manifestations make timely diagnosis of HE difficult. Herein, we report a case of HE, in which the clinical symptoms and laboratory test results mimicked viral encephalitis. A 59-year-old male patient, who presented with a fever, headache, slow and unclear speech, sentence confusion, elevated levels of anti-thyroid antibodies in the serum, an increased white blood cell count, and positivity for anti-thyroid antibodies in the CSF, was finally diagnosed with HE and responded well to a small dose of methylprednisolone. This report helps bring the attention of clinicians to the fact that HE should be considered when cases of unexplained encephalopathy are encountered.

## Background

Hashimoto’s encephalopathy (HE) is a rare neuropsychiatric syndrome associated with thyroid antibodies and was first reported by Brain in 1996 (Brain et al., 1966). HE presents with a broad range of clinical symptoms, including neurological manifestations such as stroke-like episodes, seizures, confusion, myoclonus, ataxia, tremors, and dementia, as well as psychiatric manifestations, including acute psychosis, depressive disorders, personality changes, hallucinations, and schizophrenia (Kirshner, 2014; Menon et al., 2017). In this case report, we describe a patient with HE whose clinical symptoms and laboratory test results mimicked viral encephalitis.

## Case Report

**FIGURE 1:**
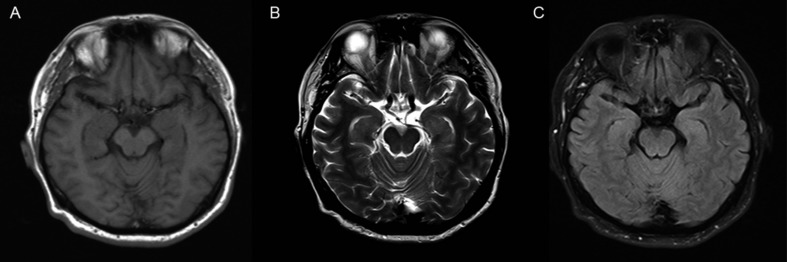
MRI imaging of brain. There are no abnormal findings in the brain MRI imaging of the patient. **(A)** T1-weighted image; **(B)** T2-weighted image; **(C)** FLAIR image.

A 59-year-old man who presented with fever, headache, and awkward speech which specifically manifested as slow and unclear speech, was admitted to the hospital. He denied recent infections such as flu or gastroenteritis, travel, and other possible reasons, which could be responsible for the fever, which peaked at 39.5°C. Previous medical history revealed gout for 20 years, but no drugs were prescribed. His neurological examination and cranial computed tomography (CT) and magnetic resonance imaging (MRI) (Figure 1) scans were normal. EEG results showed minor irregularities in waves (5–20 μv 14–20 Hz β) emitted from the bilateral hemispheres. Blood routine, C-reactive protein, serum vitamin B12, and folic acid, as well as other autoimmunity makers containing antinuclear antibody (ANA), anti-neutrophil cytoplasmic antibodies (ANCA), and rheumatoid factors were all unremarkable. The cerebrospinal fluid (CSF) showed an increased white blood cell (WBC) count (104 × 10^6^/L, reference range 0–8 × 10^6/L) and elevated protein levels (1.68 g/L, reference range 0.15–0.45 g/L). Culture, smear, and bacterial, fungal, viral, and tubercle bacillus antibodies in the serum and CSF were negative. He was diagnosed with viral encephalitis and treated with antiviral agents. His symptoms eased within a week and he was discharged from the hospital. Five months later, he was referred to our hospital again due to a fever of 38.5°C and occasional sentence confusion. Another lumbar puncture was performed; the CSF had a WBC count of 39 × 10^6/L and the protein content was 1.32 g/L. The cranial MRI and laboratory test findings were almost normal except for decreased thyroid function and increased anti-thyroid autoantibody (ATA) levels in the serum and CSF. The initial findings were as follows: in the serum, the thyroid stimulating hormone (TSH) concentration was 43.39 uIU/ml (reference range (RR): 0.27–4.2 uIU/ml), free triiodothyronine (FT3) concentration was 2.89 pmol/ml (RR: 3.1–6.8 pmol/ml), free thyroxine (FT4) concentration was 7.18 pmol/ml (RR: 12.0–22.0 pmol/ml), total triiodothyronine (TT3) concentration was 1.17 pmol/ml (RR: 1.3–3.1 pmol/ml), and total thyroxine (TT4) concentration was 44.26 pmol/ml (RR: 66–181 pmol/ml); the titer of anti-thyroglobulin autoantibodies (TgAb) was 1274 IU/ml (RR: < 115 IU/ml) and the titer of anti-thyroperoxidase autoantibodies (TPOAb) was 600 IU/ml (RR: < 35 IU/ml). In the CSF, ATA was positive (TPOAb 17.06 IU/ml, TgAb 16.02 IU/ml). Ultrasound imaging indicated diffuse lesions of the thyroid. Antibody analysis of anti-NMDAR, AMPA1, AMPA2, LGI1, CASPR2, GABA, GAD, anti-Hu, Yo, Ri, MAI, MA2, CV2, Amphiphysin, SOX-1, Tr, Zic4, and GAD65, were all negative in both the serum and CSF. The patient was ultimately diagnosed with HE, Hashimoto’s Thyroiditis, and hypothyroidism and prescribed methylprednisolone and Euthyrox. Methylprednisolone was started at a dose of 80 mg/day for 1 week and reduced to 40 mg/day in the 2 week; subsequently, oral prednisolone was prescribed, which was weaned at a rate of 5 mg per week, that is oral prednisolone was used for a total of 8 weeks. His symptoms were relieved in 3 days. The patient remained healthy during a follow up period of 1 year.

## Discussion

The estimated prevalence rate of HE is 2.1 in 100,000 and the sex ratio (female to male) is 4:1 (Ferracci et al., 2004). The course of HE may be progressive, relapsing-remitting, or even self-limiting. In the present case, the patient appeared to have a relapsing-remitting course.

Combined with the patient’s symptoms, normal MRI results, high titers of ATA in the serum and ATA positivity of the CSF, the patient was finally diagnosed with HE after excluding other potential causes including stroke, tumor, central nervous system infection, autoimmune encephalitis, and paraneoplastic syndrome.

Overall, the pathophysiology of HE is still inconclusive. On the basis of the neuropathological findings, an autoimmune vasculitis mechanism has been proposed. In a previous study, brain biopsies revealed that lymphocytic infiltration around the venules and arterioles may be involved (Duffey et al., 2003). Another possible mechanism involved is that ATAs attack antigens that are shared by the thyroid and the brain, and for this reason high titers of ATAs including TPOAb, TgAb, and anti-TSH receptor (TSH-R) antibodies in the serum, and sometimes in CSF, are considered hallmark features of HE (Yoneda, 2018). Similarly, high titers of ATA and CSF-ATA were found in our case. In a previous study, plasma exchange therapy resulted in a profound improvement of the patients’ symptoms (Tran et al., 2018). Together, these findings suggest that ATA plays an important role in HE. However, elevated ATA levels in the serum are also found within the general population and are especially common in elderly individuals. Further, the extent of ATA elevation is not related to the severity of HE. Although the patients’ serum ATA levels still remained high after methylprednisolone therapy, his symptoms were resolved (titer of anti-thyroglobulin autoantibodies (TgAb): 041 IU/ml, RR: < 115 IU/ml; titer of anti-thyroperoxidase autoantibodies (TPOA): 600 IU/ml, RR: <35 IU/ml). Our results confirm that there is no relationship between the extent of ATA elevation and the severity of HE.

Although the correlation between ATA and HE is still unclear (Kirshner, 2014), diagnostic criteria for HE were proposed by a team of experts in Lancet Neurology in 2016. The criteria are as follows: (1) encephalopathy with seizures, myoclonus, hallucinations, or stroke-like episodes; (2) subclinical or mild overt thyroid disease (usually hypothyroidism); (3) normal findings or non-specific abnormalities shown by brain MRI; (4) presence of thyroid antibodies in the serum (thyroid peroxidase, thyroglobulin); (5) absence of well-characterized neuronal antibodies in the serum and CSF; and (6) reasonable exclusion of alternative causes. HE can be diagnosed if a patient meets all the criteria (Graus et al., 2016). Our patient met all the above criteria, and he could therefore be diagnosed with HE. Most patients with HE respond well to steroids; however, when patients are steroid-resistant, other immunosuppression therapies including plasma exchange, IVIg, methotrexate, and mycophenolate have been shown to be effective. High doses of methylprednisolone (500–1000 mg) are most frequently used. Because the patient showed mild symptoms and refusal the use of high dose of methylprednisolone, we prescribed a low dose of methylprednisolone. Our patient underwent steroid therapy and has since remained healthy. The patient did not receive any other immunotherapy treatments during follow-up. In a retrospective observational study performed by Mamoudjy et al. (2013) the research results indicated that some HE patients underwent a relapse, even a third attack, which had a shorter interval time (mean 18 days) compared with that of the second attack (mean 213 days). Patients with relapses needed methylprednisolone or immunosuppressive therapy again (Mamoudjy et al., 2013). Our patient suffered one relapse about 150 days after the first attack and steroid therapy was effective. Sequelae, such as headache, memory disorders and so on, was also frequent (Mamoudjy et al., 2013), however, our patient did not have any sequelae left luckily.

Hashimoto’s encephalopathy is difficult to diagnose accurately, because of its association with a broad range of clinical symptoms, as well its non-specific neuroimaging and electroencephalogram presentation. A previous article reported a case of HE that was initially misdiagnosed as viral encephalitis (He et al., 2013), in which the patient presented with progressively impaired cognitive function and uncontrolled seizures without fever. In our case, the increased WBC count and CSF protein combined with a fever and headache lead us to diagnose it as viral encephalitis. Fever has been described in several patients with HE with and without thyroid disorders (Huang et al., 2011; Lu et al., 2015). Although the reason for fever in HE is unclear, Lu et al. (2015) suggested it may be a direct result of inflammation combined with the autoimmune vasculitis.

Hashimoto’s encephalopathy can manifest in many different ways, including stroke-like episodes, seizures, confusion, myoclonus, acute psychosis, depressive disorders, and hallucinations, causing HE to be confused with other diseases. Uwatoko et al. (2018) reported a case of a patient who presented with parkinsonism and a tumor-like lesion revealed by brain MRI. After biopsy, it was found that the lesion was not a tumor, and HE was finally confirmed (Uwatoko et al., 2018). Patients with HE can also present with unusual symptoms such as pseudobulbar palsy, sensorimotor polyneuropathy, catatonic symptoms, vertigo, muscle weakness, chorea, opsoclonus, and a trigeminal neuralgia type headache, among others (Beckmann et al., 2011; Salazar et al., 2012; Sharan et al., 2015; Ueno et al., 2016; Karthik et al., 2017; Emeksiz et al., 2018; Oz Tuncer et al., 2018). Rapidly progressive dementia, as a common manifestation of HE, makes it necessary to distinguish HE from other diseases caused by vascular, infectious, toxic-metabolic, and autoimmune factors, metastasis/neoplasia, iatrogenic/inborn errors of metabolism, neurodegenerative diseases, and systemic diseases/seizures (Paterson et al., 2012).

## Conclusion

In conclusion, the diagnosis of HE is rather complex but valuable because of its dramatic response to immunosuppressive therapy. When clinicians are faced with unexplained encephalitis, thyroid function and ATA levels should be considered as conventional tests. HE can be excluded as a diagnosis for patients with normal serum levels of ATA. For patients with increased levels of ATA, HE should be considered after ruling out other possible diseases.

## Data Availability Statement

The datasets generated for this study are available on request to the corresponding author.

## Ethics Statement

The studies involving human participants were reviewed and approved by the Ethics Committee of The First Hospital of Jilin University, China. The patients/participants provided their written informed consent to participate in this study. Written informed consent was obtained from the individual(s) for the publication of any potentially identifiable images or data included in this article.

## Author Contributions

HM contributed to the conception and design of the manuscript. MY wrote the first draft of the manuscript. YY, XM, YX, and NS wrote sections of the manuscript. All authors contributed to manuscript revision, read, and approved the submitted version.

## Conflict of Interest

The authors declare that the research was conducted in the absence of any commercial or financial relationships that could be construed as a potential conflict of interest.
